# Longitudinal assessment of the prevalence of *Fusobacterium necrophorum*, *Fusobacterium* var*ium*, and *Salmonella enterica* in the nasal cavity, ruminal fluid, and feces of finishing beef steers with and without liver abscesses

**DOI:** 10.3389/fmicb.2025.1565303

**Published:** 2025-03-20

**Authors:** Colten W. Dornbach, Paul R. Broadway, James E. Wells, Kallie D. Childress, Aubrey C. Thompson-Smith, Landon G. Canterbury, Nicole C. Burdick Sanchez, Jacque Mathieu, Cory Schwarz, Jenny Laverde Gomez, Marina Tikhonova, T. G. Nagaraja, Michael L. Galyean, Kristin E. Hales

**Affiliations:** ^1^Department of Animal and Food Sciences, Texas Tech University, Lubbock, TX, United States; ^2^USDA-ARS Livestock Issues Research Unit, Lubbock, TX, United States; ^3^USDA-ARS, U.S. Meat Animal Research Center, Clay Center, NE, United States; ^4^Department of Civil and Environmental Engineering, Rice University, Houston, TX, United States; ^5^Sentinel Environmental Group, LLC, Houston, TX, United States; ^6^College of Veterinary Medicine, Kansas State University, Manhattan, KS, United States; ^7^Department of Veterinary Sciences, Texas Tech University, Lubbock, TX, United States

**Keywords:** feedlot beef cattle, *Fusobacterium necrophorum*, *Fusobacterium* var*ium*, *Salmonella*, liver abscess

## Abstract

The objective was to longitudinally assess the prevalence of *F. necrophorum* subsp. *necrophorum*, *F. necrophorum* subsp. *funduliforme*, *F.* var*ium*, and *Salmonella enterica* in the nasal cavity, ruminal fluid, and feces of finishing beef steers with and without LA. Crossbred steers (*n* = 225; 353 ± 39.6 kg) were transported to a feedlot and fed a high-concentrate diet. Nasal, ruminal fluid, and fecal samples were collected following feedlot arrival (d 5), 1 week after adaptation to a finishing diet (d 35), and the day before harvest (study end). Livers were collected at harvest and examined for LA, and cattle were subsequently assigned into either control or liver abscess groups. Overall LA prevalence was 18.7%. The concentration and prevalence of *Salmonella* decreased in ruminal fluid and increased in feces with days on feed (*p* < 0.01). Conversely, ruminal fluid prevalence of *F. necrophorum* subsp. *necrophorum* and *F.* var*ium* increased with days on feed (*p* < 0.01). *Fusobacterium* abundance in ruminal fluid and feces was not indicative of LA development except for *F. varium* being more abundant in the ruminal fluid of steers with LA (*p* < 0.01). Abundance of *F. necrophorum* subsp. *necrophorum* was greater in abscessed liver tissue than healthy tissue (*p* = 0.03), although no other differences in bacterial abundance or prevalence were observed in livers. Overall, *Fusobacterium* and *Salmonella* prevalence in the nasal cavity, ruminal fluid, and feces were affected by days on feed, but their prevalence and abundance were not indicative of LA occurrence.

## 1 Introduction

Liver abscesses (LA) in finishing beef cattle are a significant economic concern for the feedlot industry because of decreased body weights and hot carcass weights (Brink et al., 1990; Brown and Lawrence, 2010), contributing to an estimated economic burden of almost $1 billion (Lawrence, 2024). On an individual-pen basis, LA prevalence ranges from 0 to 95.5%, with the overall prevalence increasing since 2012 (Grimes et al., 2024). Liver abscesses are complex, polymicrobial, and involve multiple organs (Broadway et al., 2024). The bacterial etiology of LA has been extensively studied, with *Fusobacterium necrophorum* subsp. *necrophorum* considered the primary causative agent (Amachawadi and Nagaraja, 2016; Pinnell and Morley, 2022; McDaniel et al., 2024a).

Historically considered a normal resident of the bovine gastrointestinal tract (GIT; Jang and Hirsh, 1994; Langworth, 1977), *F. necrophorum* subsp. *necrophorum* is an opportunistic pathogen commonly isolated in both necrotic respiratory infections (Seimiya et al., 2004; Tadepalli et al., 2009) and LA (Nagaraja and Chengappa, 1998). Recently, identification of *F* var*ium* as the dominant species of *Fusobacterium* in the rumen of cattle has called into question the validity of previous culture-dependent methods (Schwarz et al., 2023; Deters et al., 2024a). Prevalence of *F. necrophorum* subsp. *necrophorum* in LA ranges from 71 to 100% (Herrick et al., 2022; Lechtenberg et al., 1988; Nagaraja and Chengappa, 1998). The common theory on etiology of LA suggests acidosis-induced rumenitis allows for bacterial invasion and colonization of the ruminal wall (Jensen et al., 1954; Nagaraja and Lechtenberg, 2007), thereby increasing bacterial translocation into portal vein circulation (Tadepalli et al., 2009). Once *F. necrophorum* subsp. *necrophorum* is translocated to the liver, leukotoxins and endotoxins protect it from phagocytosis (Emery et al., 1985; Tan et al., 1996) and induce hepatocyte-mediated apoptosis (Amachawadi and Nagaraja, 2016).

Recently, *Salmonella enterica* (denoted as *Salmonella*) has been isolated from LA (Amachawadi and Nagaraja, 2015; Amachawadi et al., 2017) at a prevalence of 27.5% nationally and 23.8% in association with *F. necrophorum* (Herrick et al., 2022). Nonetheless, in the High Plains cattle feeding region, the incidence of *Salmonella* alone or with *F. necrophorum* increases to 84.6 and 76.7%, respectively (Herrick et al., 2022). Hind-gut acidosis or stress-induced inflammation can increase the translocation of *Salmonella* across the intestinal epithelium, where *Salmonella* actively infects phagocytic and non-phagocytic cells (Ibarra and Steele-Mortimer, 2009; Sanz-Fernandez et al., 2020). Thus, the lymphatic system could provide another pathway for *Salmonella* to enter the liver besides portal vein circulation. Currently, little data substantiates the role of *Salmonella* in LA formation or the concentration and prevalence of *Fusobacterium* and *Salmonella* throughout the GIT in relation to LA occurrence. Therefore, we hypothesize that bacterial populations associated with LA will differ in the GIT of beef steers with and without LA. Our objective was to longitudinally assess the prevalence of *F. necrophorum* subsp. *necrophorum*, *F. necrophorum* subsp. *funduliforme, F.* var*ium*, and *Salmonella enterica* in the nasal cavity, ruminal fluid, and feces of finishing beef steers with and without LA.

## 2 Materials and methods

All experimental procedures were approved by the Texas Tech University Institutional Animal Care and Use Committee (approval number 2022–1273) and conducted from May 2023 to February 2024.

### 2.1 Animal management

Crossbred steers (*n* = 225; 353 ± 39.6 kg) were sourced from the Texas Panhandle, transported to the Burnett Center for Research and Instruction, and blocked by arrival group into 2 source blocks. On d 0, steers were received in soil-surface, partially shaded outdoor pens (4.9 m × 30 m), administered vaccinations (Myco-B One Dose, American Animal Health, Fort Worth, TX; Bovilis Vista 5Q, Merck, Rahway, NJ; Bovilis Vision 7 with Spur, Merck; I-site XP, Huvepharma, Peachtree City, GA), anthelmintic (Cydectin; Elanco, Indianapolis, IN), and received a Revalor-XS implant (200 mg of trenbolone acetate +40 mg of estradiol 17β; Merck). On d 21, steers were moved into concrete, slatted-floor pens. From d 0 to study end (d 250 for block 1 and d 221 for block 2), steers were fed a standard grain-based finishing diet representative of those fed in the High Plains region (Table 1). At study end, steers were harvested at a commercial abattoir.

**Table 1:** Ingredient composition of diets fed to feedlot beef steers from d 0 to study end.

	Receiving	Transition 1	Transition 2	Finishing
Item	d 0 to 7	d 8 to 14	d 15 to 21	d 22 to End^1^
Ingredient, % DM				
Steam-flaked corn	17.84	43.61	60.00	64.05
Sweet bran^2^	56.61	33.96	20.98	24.44
Alfalfa hay	21.86	18.08	13.59	6.88
Supplement^3^	1.96	2.41	2.38	2.08
Limestone	1.73	1.94	1.93	1.82
Urea	–	–	1.12	0.73

^1^Study end was on d 250 for block 1 steers and d 221 for block 2 steers. ^2^Branded wet corn gluten feed (Blair, NE). ^3^Vitamins and minerals exceeded NASEM (2016) requirements for finishing beef steers. Rumensin 90 (Elanco, Greenfield, IN) was included at 330 mg/kg.

### 2.2 Sample collection

Nasal, ruminal fluid, and fecal samples were aseptically collected after feedlot arrival (d 5), 1 week after adaptation to the finishing diet (d 35), and the day before harvest (study end). Nasal samples (*Salmonella* only) were collected via a 5-in, rayon-tipped bacteriology swab (Fisher, Waltham, MA), whereas ruminal fluid was collected using a speculum and flexible tubing passed through the esophagus to the rumen, and feces were collected by rectal palpation. All samples were subsequently processed at the USDA-ARS Livestock Issues Research Unit (LIRU). At the commercial abattoir, LA prevalence was recorded by trained personnel from the Beef Carcass Research Center at West Texas A&M University. A 100-g sample of healthy and abscessed livers were collected before carcass chilling, placed in a sealable bag, and transported to LIRU. Liver samples were immediately emulsified using a blender for bacterial processing.

### 2.3 Sample processing for *Salmonella enterica* concentration and prevalence

Analysis of samples for *Salmonella* was conducted as described by Dornbach et al. (2023). To determine ruminal fluid, fecal, and liver *Salmonella* concentration and prevalence, 25 g of sample were diluted 1:10 in phosphate buffered saline (PBS) in a lateral filtered stomacher bag (Seward Ltd.; West Sussex, United Kingdom) and homogenized (Stomacher® 400 Circulator; Seward Ltd.) for 2 min at 230 rpm. For nasal *Salmonella* prevalence, swabs were suspended in 4.5 mL of PBS and vortexed. *Salmonella* concentrations were determined via spiral plating (Eddy Jet 2 W, Neutec Group Inc., Farmingdale, NY) 100 μL of homogenate onto xylose lysine deoxycholate (XLD; Becton, Dickinson and Co., Franklin Lakes, NJ) agar containing novobiocin (25 μg/mL). Additionally, 1 mL of homogenate was enriched in a 1:10 dilution of Tetrathionate Hajna (Remel, San Diego, CA) broth with iodine and incubated overnight at 37°C. Likewise, 1 mL of homogenate was enriched in Rappaport-Vassiliadis (Oxoid Ltd., Basingstoke, UK) broth and incubated at 42°C overnight. After incubation, enrichment broths were vortexed, and a 10-μL loop was used to streak enriched cultures onto XLD agar containing novobiocin (25 μg/mL). Following overnight incubation at 37°C and an additional 24 h at 25°C, phenotypic colonies were subjected to latex agglutination (*Salmonella* Test Kit; Oxoid Ltd) and confirmed by PCR using the *inv*A gene (Rahn et al., 1992). Assay running conditions included an initial incubation at 95°C for 1 min, followed by 35 cycles of 95°C for 30 s, 64°C for 30 s, and 72°C for 30 s. Following the last cycle there was a 4 min incubation at 72°C. Assays were performed using a Bio-Rad C1000 Thermal Cycler (Hercules, CA).

### 2.4 Sample processing for *Fusobacterium necrophorum* and *Fusobacterium* var*ium* abundance and prevalence

To determine the absolute abundance (i.e., copies/g) and prevalence (i.e., %) of ruminal fluid *F. necrophorum* and *F. varium*, dimethyl sulfoxide (DMSO) was added to a final concentration of 5% v/v, while fecal and liver samples were diluted 1:1 in PBS containing 10% DMSO. All samples were subsequently homogenized and frozen at-80°C. Frozen samples were then transported on dry ice to Sentinel Environmental in Houston, TX for analysis.

Ruminal fluid, fecal, and liver DNA were extracted using the ZymoBIOMICS™ 96 MagBead DNA kit (D4308-E, Zymo Research Corp. United States) and an OpenTrons OT-2 liquid-handling robot running a custom python script. Briefly, samples were partially thawed at room temperature to transfer 100 mg of sample into ZR BashingBead™ Lysis Tubes containing 375 μL ZymoBIOMICS™ Lysis Solution, 375 μL of DNA/RNA Shield™, and 1×10^6^ copies of lambda phage genome (N3011S; New England Biolabs, Ipswich, MA). Following mechanical lysis with a Biospec Mini-Bead Beater-16 (BioSpec Products, Inc., Bartlesville, OK), samples were transferred to a 96-deep-well plate. Extraction of DNA was conducted with the OpenTrons OT-2. As described by Deters et al. (2024a), the qPCR primers and probes used were designed to target *hgdA* for *F. necrophorum* (*hgdA*-*n*) and *F.* var*ium* (*hgdA-v*), as well as the leukotoxin promotor region, *lktA*-*n* for *F. necrophorum* subsp. *necrophorum* and *lktA*-*f* for *F. necrophorum* subsp. *funduliforme* (Table 2). Probe concentrations were optimized for each target gene. Assay running conditions were 95°C for 5 min followed by 45 cycles of 95°C for 15 s and 60°C for 40 s. Assays were performed using a Bio-Rad CFX96 Real-Time System. Lambda phage DNA was quantified to assess efficiency of extraction using primers and probes as described by Beller et al. (2002). Any sample with ≥10^2^ copies/g of *Fusobacterium* were considered positive for prevalence.

**Table 2:** Species and subspecies of *Fusobacterium*, genes targeted, and primer and probe sequences used in the qPCR assay.

Target species and subspecies	Gene target	Primer sequence (5′-3′)	Probe sequence (5′-3′)	Amplicon size
*Fusobacterium necrophorum* subsp. *necrophorum*	*lktA*-n	Forward: GCTTTGGAAGAAGCCAAACA	FAM- TGGAATCATTCCAGTAGATGGAAAAG-ZEN™/3’IB®	93 bp
		Reverse: AATGCTTCCATTCGGATTCA		
*Fusobacterium necrophorum* subsp. *funduliforme*	*lktA*-f	Forward: AAAGACGCTCAAAATAGCAAAGTT	MAX- TTGTTCCACAACAGGATGGGAGTA-ZEN™/3’IB®	80 bp
		Reverse: TTTGGATTCAACGGAATCTTG		
*Fusobacterium necrophorum*	*hgdA*-fn	Forward: CTTTTTCCAATACGGTAGATACTCC	5’TexasRed-X-TGGATTATTTGATTGGACAGTTCGA-Iowa Black RQ	94 bp
		Reverse: CCTGTCAATTCTTCCAACTGC		
*Fusobacterium varium*	*hgdA*-fv	Forward: TTCAAATACAGTGGATACACCAGAA	Cy5-AGTGGATTATCTAATCGGACAATTTGA-Iowa Black RQ	84 bp
		Reverse: AATTCTTCTAATTGTTTGATTGCATAA		
lambda phage		Forward: ACGCCACGCGGGATG	TXRed-X-ACCTGTGGCATTTGTGCTGCCG-Iowa Black RQ	
		Reverse: AGAGACACGAAACGCCGTTC		

### 2.5 Statistical analyses

The experimental design was a randomized complete block with individual steer as the experimental unit. The GLIMMIX and MIXED procedures of SAS 9.4 (SAS Inst., Cary, NC) were used to evaluate binomial and continuous data, respectively, with fixed effects of treatment, sampling time, and treatment × sampling time interaction. The interaction of block × treatment × sampling time was included as a random effect. Sampling time was the repeated measure and individual steer within block was the subject. The Kenward Roger adjustment was used to correct the degrees of freedom because of unequal treatment numbers. The covariance structure autoregressive (1) was used based on evaluation of the Akaike’s information criterion. Least squares means were separated using the Tukey option in the LSMEANS statement of SAS. Outliers were identified using the Cook’s D outlier test; ruminal fluid data for 1 steer at study end was omitted using these criteria. A *p* ≤ 0.05 was considered significant and tendencies were discussed at 0.05 < *p* ≤ 0.10.

## 3 Results

### 3.1 *Salmonella enterica* concentration and prevalence in the nasal cavity, ruminal fluid, feces, and liver

Overall LA prevalence was 18.7% (*n* = 42). No treatment × sampling time interactions were observed throughout the study (*p* ≥ 0.14). Nasal *Salmonella* concentration did not differ between steers with or without LA (*p* = 0.85; Table 3) or by collection day (*p* = 0.50). Moreover, no differences in ruminal fluid *Salmonella* concentration were observed between steers with or without LA (*p* = 0.37); however, ruminal fluid *Salmonella* concentration decreased from feedlot arrival to harvest (*p* < 0.01). Conversely, fecal *Salmonella* concentration was greatest before harvest (*p* < 0.01) and tended to be 5.9% greater in steers without LA (*p* = 0.07). Liver *Salmonella* concentrations were not affected by LA presence (*p* = 0.18; Table 4).

**Table 3:** Concentration of *Salmonella enterica* in the nasal cavity, ruminal fluid, and feces of finishing beef steers with and without liver abscesses.^1^

Item	Liver abscesses			*p*-value^2^		
	Not present	Present	SEM^3^	Trt	Time	Trt × Time
*n*	183	42				
Nasal cavity, log_10_ CFU/g						
d 5	3.16 (20/183)	3.16 (5/42)	0.096	0.85	0.50	0.93
d 35	3.06 (10/183)	3.12 (3/42)				
End^4^	3.06 (10/183)	3.05 (2/42)				
Ruminal fluid, log_10_ CFU/g						
d 5	3.61 (71/183)	3.83 (21/43)	0.200	0.37	<0.01	0.22
d 35	3.41 (59/183)	3.39 (12/43)				
End^4^	3.16 (22/183)	3.13 (4/43)				
Feces, log_10_ CFU/g						
d 5	3.48 (60/183)	3.50 (13/43)	0.035	0.07	<0.01	0.14
d 35	4.62 (118/183)	4.47 (24/43)				
End^4^	4.96 (130/183)	4.36 (25/43)				

^1Numbers in parentheses are the number of enumerable samples and total samples. 2Trt = treatment effect; Trt × Time = treatment × sampling time effect. 3Standard error of the mean. 4Study end was on d 250 for block 1 steers and d 221 for block 2 steers.^

**Table 4:** Concentration and prevalence of *Salmonella enterica* in the livers of feedlot beef steers with and without liver abscesses.^1^

Item	Liver abscesses			*P*-value^2^
	Not present	Present	SEM^3^	Trt
*n*	183	42		
*Salmonella* concentration, log_10_ CFU/g	3.02 (4/183)	3.08 (1/42)	0.035	0.18
*Salmonella* prevalence, %	6.5	9.8	3.91	0.38

^1Numbers in parentheses are the number of enumerable samples and total samples. 2Trt, treatment effect. 3Standard error of the mean.^

**Figure 1:**
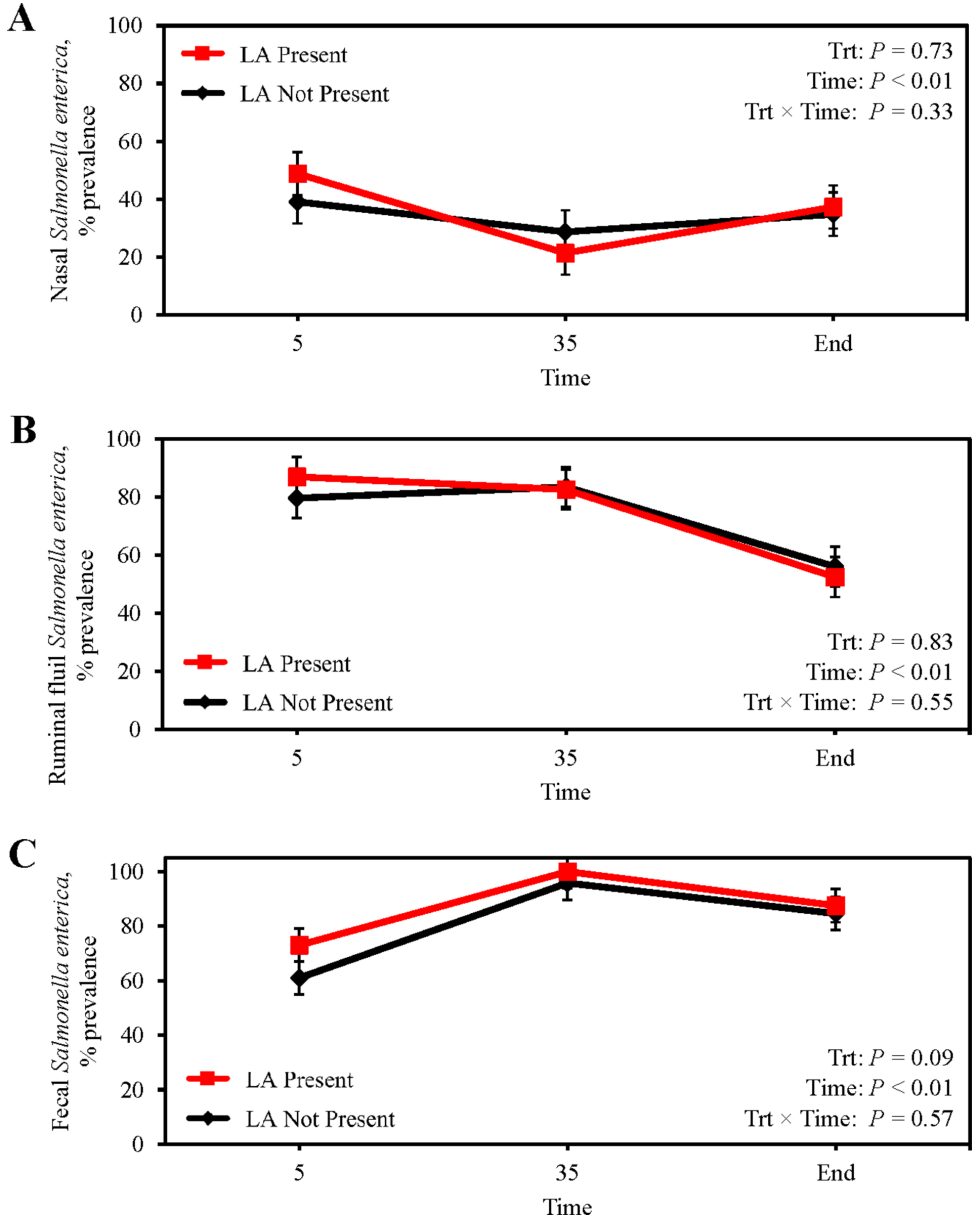
Prevalence of *Salmonella enterica* in the nasal cavity **(A)**, ruminal fluid **(B)**, and feces **(C)** of finishing beef steers with (Present; solid red line with square marker) and without (Not Present; solid black line with diamond marker) liver abscesses (LA). Samples were aseptically collected after feedlot arrival (d 5), 1 week after adaptation to the finishing diet (d 35), and the day before harvest (study end). Study end was on d 250 for block 1 steers and d 221 for block 2 steers. Error bars represent standard error of the mean. Trt = treatment effect; Trt × Time = treatment × sampling time effect.

Mean nasal *Salmonella* prevalence was 34.5%, being greatest at feedlot arrival and least on d 35 (*p* < 0.01; Figure 1A), although prevalence was not indicative of the presence of LA (*p* = 0.73). Ruminal fluid *Salmonella* prevalence did not differ between steers with or without LA (*p* = 0.83; Figure 1B) whereby mean ruminal fluid *Salmonella* prevalence was 73.2%, with prevalence decreasing from 83.3% on d 35 to 55.3% at harvest (*p* < 0.01). Fecal *Salmonella* prevalence increased from 63.2% on d 5 to 96.6% by d 35 after transition to the finishing diet (*p* < 0.01; Figure 1C), with a mean fecal *Salmonella* prevalence of 81.7%. Fecal *Salmonella* prevalence tended to be 6.4% greater in steers with LA (*p* = 0.09), and liver *Salmonella* prevalence was 9.8 and 6.5% for steers with and without LA, respectively, but did not differ between groups (*p* = 0.47; Table 4).

### 3.2 Absolute abundance and prevalence of *Fusobacterium necrophorum* and *Fusobacterium* var*ium* in ruminal fluid

**Figure 2:**
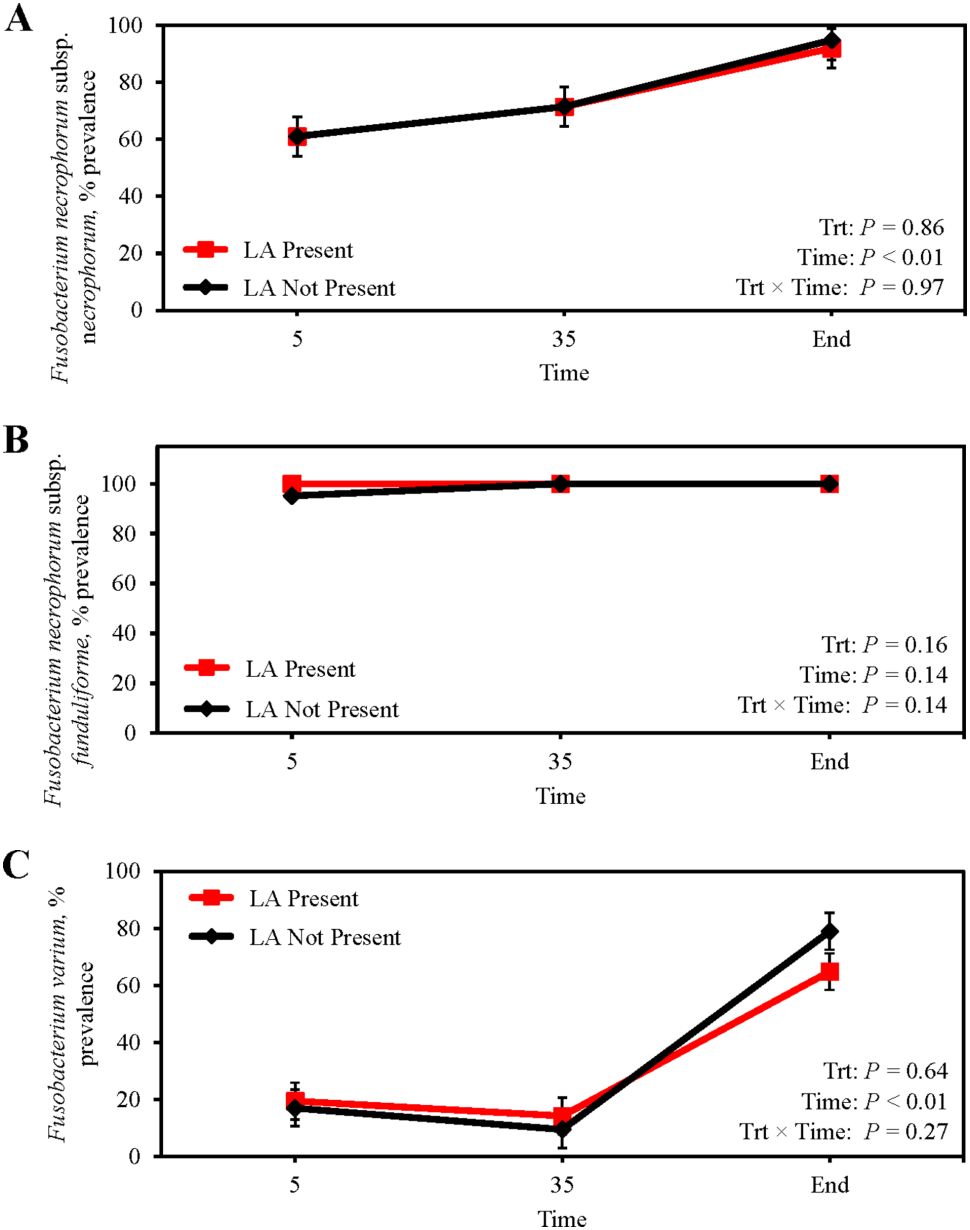
Prevalence of *Fusobacterium necrophorum* subsp. n*ecrophorum* **(A)** *Fusobacterium necrophorum* subsp. *funduliforme* **(B)** and *Fusobacterium varium* **(C)** in the ruminal fluid of finishing beef steers with (Present; solid red line with square marker) and without (Not Present; solid black line with diamond marker) liver abscesses (LA). Samples were aseptically collected after feedlot arrival (d 5), 1 week after adaptation to the finishing diet (d 35), and the day before harvest (study end). Study end was on d 250 for block 1 steers and d 221 for block 2 steers. Error bars represent standard error of the mean. Trt, treatment effect; Trt × Time = treatment × sampling time effect.

Absolute abundance of *F. necrophorum* subsp. *necrophorum* (*p* = 0.38; Table 5) and subsp. *funduliforme* (*p* = 0.23) in ruminal fluid was not different between steers with or without a LA. Similarly, *F. necrophorum* subsp. *necrophorum* (*p* = 0.32) and subsp. *funduliforme* (*p* = 0.79) abundance in ruminal fluid did not change from feedlot arrival to harvest. Conversely, the abundance of *F.* var*ium* in the ruminal fluid of steers with a LA was 192% greater than those without a LA (*p* < 0.01), although no differences across collection day were observed (*p* = 0.44). Ruminal fluid prevalence of *F. necrophorum* subsp. *necrophorum,* subsp. *funduliforme,* and *F.* var*ium* were not suggestive of LA formation (*p* ≥ 0.16; Figures 2A–C). From feedlot arrival to harvest, *F. necrophorum* subsp. *necrophorum* and *F.* var*ium* prevalence increased 54.4 and 300%, respectively (*p* < 0.01) regardless of LA prevalence. Mean *F. necrophorum* subsp. *funduliforme* prevalence in ruminal fluid was 99.2% and did not differ across collection day (*p* = 0.14).

**Table 5:** Absolute abundance (copies/g) of *Fusobacterium necrophorum* subsp. *necrophorum, F. necrophorum* subsp. *funduliforme,* and *F. varium* in the ruminal fluid of feedlot beef steers with and without liver abscesses.

Item	Liver abscesses			*P*-value^1^		
	Not present	Present	SEM^2^	Trt	Time	Trt × Time
*n*	42	42				
*Fusobacterium necrophorum* subsp. *necrophorum*						
d 5	1.97 × 10^6^	4.04 × 10^6^	1.866 × 10^6^	0.38	0.32	0.85
d 35	5.69 × 10^5^	7.35 × 10^5^				
End^3^	2.07 × 10^6^	3.64 × 10^6^				
*Fusobacterium necrophorum* subsp. *funduliforme*						
d 5	1.04 × 10^7^	9.03 × 10^6^	1.148 × 10^7^	0.23	0.79	0.42
d 35	1.82 × 10^6^	2.98 × 10^7^				
End^3^	6.93 × 10^6^	8.52 × 10^6^				
*Fusobacterium varium*						
d 5	4.52 × 10^2^	9.05 × 10^7^	3.667 × 10^7^	<0.01	0.44	0.41
d 35	8.25 × 10^4^	1.50 × 10^6^				
End^3^	1.82 × 10^6^	9.99 × 10^4^				

^1Trt = treatment effect; Trt × Time = treatment × sampling time effect. 2Standard error of the mean. 3Study end was on d 250 for block 1 steers and d 221 for block 2 steers.^

### 3.3 Absolute abundance and prevalence of *Fusobacterium necrophorum* and *Fusobacterium varium* in feces

**Figure 3:**
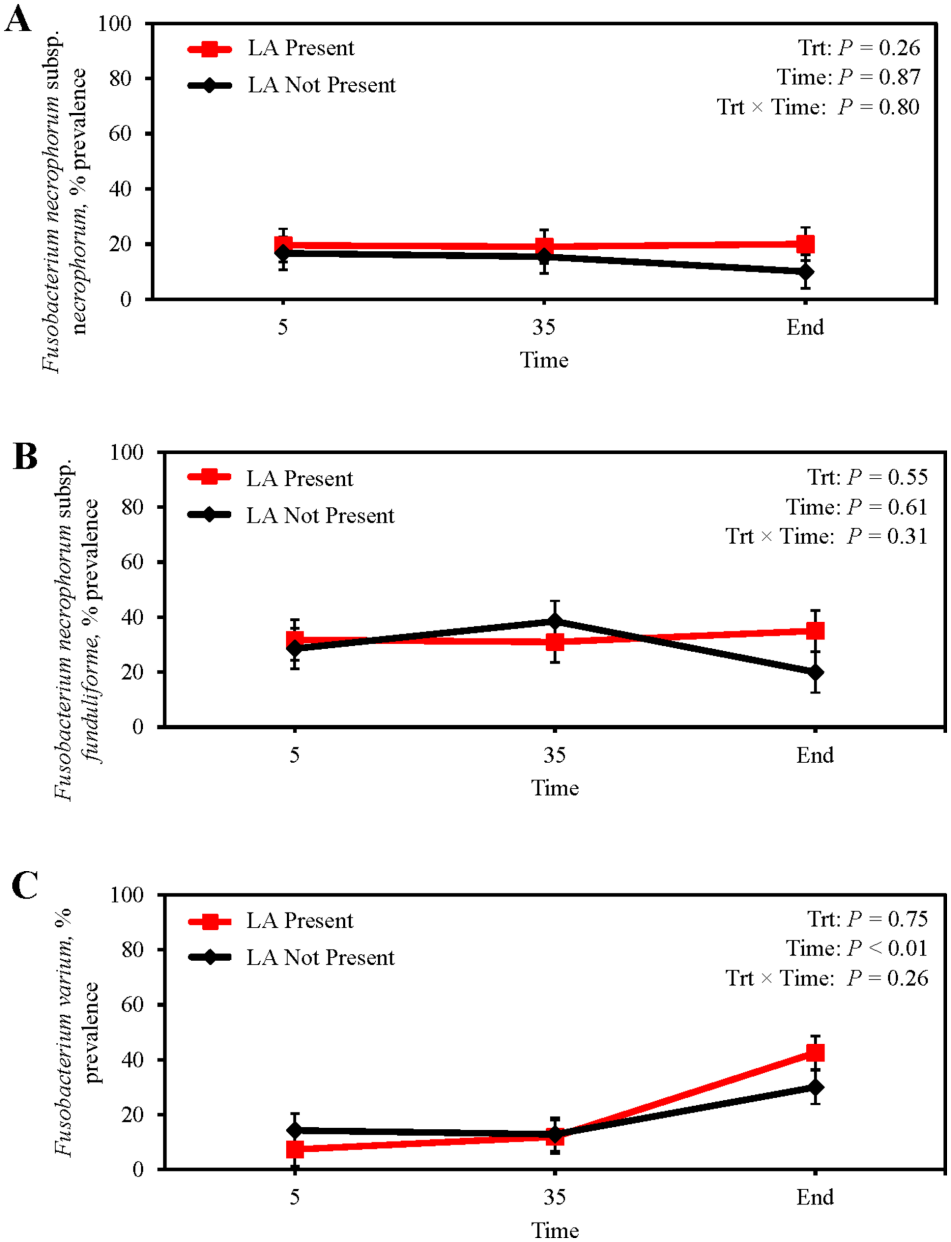
Prevalence of *Fusobacterium necrophorum* subsp. n*ecrophorum* **(A)** *Fusobacterium necrophorum* subsp. *funduliforme* **(B)** and *Fusobacterium varium* **(C)** in the feces of finishing beef steers with (Present; solid red line with square marker) and without (Not Present; solid black line with diamond marker) liver abscesses (LA). Samples were aseptically collected after feedlot arrival (d 5), 1 week after adaptation to the finishing diet (d 35), and the day before harvest (study end). Study end was on d 250 for block 1 steers and d 221 for block 2 steers. Error bars represent standard error of the mean. Trt = treatment effect; Trt × Time = treatment × sampling time effect.

Absolute abundance of *F. necrophorum* subsp. *necrophorum* in feces did not differ between steers with or without a LA (*p* = 0.28; Table 6) or from feedlot arrival to harvest (*p* = 0.32). Similarly, fecal abundance of *F. necrophorum* subsp. *funduliforme* did not differ with the presence of LA (*p* = 0.19) or longitudinally (*p* = 0.49). *Fusobacterium* var*ium* tended to have a greater abundance in the feces of steers without a LA (*p* = 0.10), although no differences were observed across sampling days (*p* = 0.35). The presence of LA at harvest was not attributable to differences in the fecal prevalence of *F. necrophorum* subsp. *necrophorum,* subsp. *funduliforme,* and *F. varium* (*p* ≥ 0.26; Figures 3A–C). Mean fecal *F. necrophorum* subsp. *necrophorum* and subsp. *funduliforme* prevalence was 16.8 and 30.8%, respectively, and did not differ from feedlot arrival to harvest (*p* ≥ 0.61). Fecal *F. varium* prevalence increased from 10.8% at feedlot arrival to 36.3% at harvest (*p* < 0.01).

**Table 6:** Absolute abundance (copies/g) of *Fusobacterium necrophorum* subsp. *necrophorum*, *F. necrophorum* subsp. *funduliforme*, and *F. varium* in the feces of feedlot beef steers with and without liver abscesses.

Item	Liver abscesses			*P*-value^1^		
	Not present	Present	SEM^2^	Trt	Time	Trt × Time
*n*	42	42				
*Fusobacterium necrophorum* subsp. *necrophorum*						
d 5	2.09 × 10^4^	1.34 × 10^7^	5.196 × 10^6^	0.28	0.32	0.32
d 35	9.39 × 10^4^	1.56 × 10^5^				
End^3^	1.81 × 10^2^	7.62 × 10^4^				
*Fusobacterium necrophorum* subsp. *funduliforme*						
d 5	2.83×10^3^	1.01 × 10^5^	3.846 × 10^4^	0.19	0.49	0.41
d 35	6.88 × 10^3^	1.13 × 10^4^				
End^3^	9.72 × 10^3^	2.81 × 10^4^				
*Fusobacterium varium*						
d 5	6.21 × 10^4^	5.23 × 10^4^	1.184 × 10^5^	0.10	0.35	0.26
d 35	3.86 × 10^5^	1.96 × 10^3^				
End^3^	1.18 × 10^4^	1.01 × 10^4^				

^1Trt = treatment effect; Trt × Time = treatment × sampling time effect. 2Standard error of the mean. 3Study end was on d 250 for block 1 steers and d 221 for block 2 steers.^

3.4 Absolute abundance and prevalence of *F. necrophorum* and *F. varium* in livers.

Although liver *F. necrophorum* subsp. *necrophorum* prevalence was not affected by the presence of an abscess (*p* = 0.67; Table 7), *F. necrophorum* subsp. *necrophorum* abundance was 195% greater for steers with a LA compared with those without a LA (*p* = 0.03). The abundance (*p* ≥ 0.20) and prevalence (*p* ≥ 0.65) of *F. necrophorum* subsp. *funduliforme* and *F. varium* in liver were not affected by the presence of an abscess.

**Table 7:** Absolute abundance and prevalence of *Fusobacterium necrophorum* subsp. *necrophorum, F. necrophorum* subsp. *funduliforme*, and *F. varium* in the livers of feedlot beef steers with and without liver abscesses.

Item	Liver abscesses			*P*-value
	Not present	Present	SEM^1^	Trt
*n*	42	42		
Absolute abundance, copies/g				
*Fusobacterium necrophorum* subsp. *necrophorum*	8.33 × 10^5^	6.13 × 10^7^	1.983 × 10^7^	0.03
*Fusobacterium necrophorum* subsp. *funduliforme*	6.96 × 10^4^	5.17 × 10^4^	3.397 × 10^7^	0.29
*Fusobacterium varium*	3.16 × 10^2^	2.66 × 10^3^	1.273 × 10^3^	0.20
Prevalence, %				
*Fusobacterium necrophorum* subsp. *necrophorum*	50.00	54.76	7.791	0.67
*Fusobacterium necrophorum* subsp. *funduliforme*	40.48	42.86	7.697	0.83
*Fusobacterium varium*	4.76	7.14	3.690	0.65

^a,b,cMeans with different superscripts in same row differ, p ≤ 0.05. 1Standard error of the mean.^

## 4 Discussion

Liver abscesses are frequently described as a polymicrobial infection, with most studies concluding that *F. necrophorum* is the primary causative agent (Broadway et al., 2024). Previous research has supported a causal link between acidosis-induced rumenitis and LA through increased bacterial translocation into portal vein circulation (Smith, 1944; Jensen et al., 1954; Nagaraja and Chengappa, 1998; Tadepalli et al., 2009). Nonetheless, venous drainage is not limited to the rumen, draining the entire GIT and associated visceral organs. Although a few studies have isolated *Salmonella* from LA (Amachawadi and Nagaraja, 2015; Amachawadi et al., 2017; Herrick et al., 2022), no literature has directly investigated a link between the presence of LA and *Salmonella* in the GIT.

### 4.1 *Salmonella enterica* in the nasal cavity, ruminal fluid, feces, and liver

*Salmonella* transmission primarily occurs through direct fecal-oral contamination (e.g., from other cattle, rodents, or birds) or indirectly through contaminated feed consumption (Cho et al., 2006; Stevens et al., 2009). Transdermal and intranasal *Salmonella* infection have also been recorded (Fedorka-Cray et al., 1995; Olafson et al., 2016). In dairy calves experiencing salmonellosis, 18.2% (4/22) of nasal secretions were positive for *S*. Typhimurium (Nolan et al., 1995). Moreover, dairy calves intranasally inoculated with *S.* Dublin (1.8 × 10^6^ cells/calf) had positive nasal mucosal secretions for up to 9 d after inoculation and positive feces up to 14 d after inoculation (Nazer and Osborne, 1977). In the current study, nasal *Salmonella* concentrations remained low from feedlot arrival to harvest and were not indicative of LA presence. Shortly after steers were received in soil-surfaced pens, nasal *Salmonella* prevalence was 40.9% (Figure 1A); however, after placement onto clean, concrete slated-floor pens, prevalence decreased by 33.3%. Movement of cattle into concrete slated-floor pens likely lessened *Salmonella* reinfection. Miller et al. (2008) reported an increase in *Salmonella* enumeration on the hides of cattle exposed to dust. Nevertheless, from d 35 to study end, nasal *Salmonella* prevalence increased 29.3% despite the final collection occurring in winter. Allan et al. (2004) reported that *Salmonella* survival on biologically contaminated concrete surfaces was greater compared with non-contaminated concrete surfaces at either 4°C or 10°C. Therefore, accumulation of contaminated feces after 200 d in concrete pens and cross contamination with vectors, like birds and rodents, consuming feed from feed bunks during winter (Gwenzi et al., 2021) increased the likelihood of inhaling *Salmonella*.

*Salmonella* concentrations and prevalence in ruminal fluid were not associated with LA presence at harvest. *Salmonella* has previously been isolated from ruminal fluid at harvest, with prevalence ranging from 0.3 to 91% (Grau et al., 1968; Van Donkersgoed et al., 1999; McEvoy et al., 2003; Fegan et al., 2005). Previous literature attributed the variation in ruminal fluid *Salmonella* isolation to differences in ruminal pH and volatile fatty acid (VFA) concentrations, which are believed to exert bacteriostatic and bactericidal effects on *Salmonella* (Mattila et al., 1988; Corrier et al., 1990). To the authors’ knowledge, this study was the first to longitudinally assess *Salmonella* in the ruminal fluid of finishing beef cattle (Figure 1B). The decrease in both *Salmonella* concentration and prevalence with days on feed supports the previous notion that the rumen becomes an unfavorable environment for *Salmonella* persistence and multiplication as total VFA concentrations presumably increase with time on feed. From d 0 to 7, cattle received a high-forage receiving diet before being transitioned to a high-concentrate finishing diet by d 22. Although we did not measure ruminal VFA concentrations in the current study, Penner et al. (2009) reported total VFA concentrations increase, while ruminal pH concurrently decreases, in cattle that are consuming a high-concentrate diet.

In contrast to ruminal fluid, fecal *Salmonella* concentrations increased with days on feed, likely reflecting the colonization of *Salmonella* in the lower GIT where fermentative activity is more limited than in the rumen (Bolton et al., 2011). From feedlot arrival to harvest, fecal *Salmonella* concentrations increased 39.3% regardless of LA presence (Figure 1C). Jennings et al. (2021) previously reported that *Salmonella* incidence within ileal and colonic epithelial tissues increased with days on feed. From d 5 to 35, fecal *Salmonella* prevalence increased, corresponding to sample collections from late May to early August. Seasonality can partially explain this phenomenon as warmer seasons are favorable for *Salmonella* persistence in feedlots compared with colder seasons (Barkocy-Gallagher et al., 2003; Webb et al., 2017; Wottlin et al., 2022). Greater temperatures during summer can induce heat stress, resulting in feed intake disruptions and inflammation that increase *Salmonella* colonization of the GIT. Likavec et al. (2016) noted *Salmonella* incidence increased 54% for every 5°C increase in average temperature and 29% for every 5-unit increase in the temperature humidity index. Temperature stability is also important for long-term *Salmonella* survival in soil and manure (Holley et al., 2006; Semenov et al., 2007). Large fluctuations in manure or soil temperature, such as freeze–thaw cycles associated with colder months, can rapidly decrease environmental *Salmonella* concentrations (Semenov et al., 2007). This might also explain the decrease in fecal *Salmonella* prevalence from d 35 to harvest as cattle were harvested in winter when environmental temperature fluctuated between-14°C to 24°C.

From feedlot arrival to harvest, fecal *Salmonella* concentration tended to be greater in steers without LA, whereas *Salmonella* prevalence was greater in steers with LA. In the small intestine, *Salmonella* can either be endocytosed by M cells located in Peyer’s patches or induce cytoskeletal changes in epithelial cells, leading to membrane ruffling and bacterial internalization (Lostroh and Lee, 2001). Entry into cells can result in a pro-inflammatory response and uptake by macrophages and neutrophils (Clark et al., 1994; Johansson et al., 2006). Survival and replication within macrophages are essential for entry and persistence in lymph nodes and eventual liver colonization (Watson and Holden, 2010; Ilyas et al., 2017). Although not measured directly in the current study, the greater fecal *Salmonella* concentrations coupled with lesser fecal *Salmonella* prevalence could suggest cattle without LA had improved lower GIT epithelial integrity. In dairy steers ruminally inoculated with both *F. necrophorum* and *S*. Lubbock, 60% of ruminal and ileal tissue samples were positive for *Salmonella* (McDaniel et al., 2024b). While information regarding a synergistic relationship between *Salmonella* and *F. necrophorum* remains elusive, greater LA severity and prevalence has been noted when *Salmonella* and *F. necrophorum* are cultured in combination from LA (Herrick et al., 2022; McDaniel et al., 2024b). Further studies are warranted to validate these findings and determine whether *Salmonella* acts as a primary pathogen contributing to LA development or if its presence in the GIT facilitates *Fusobacterium* entry into the portal circulation.

Nationally, *Salmonella* has been isolated from 33.3% of LA from fed beef steers, with regional prevalence ranging from 0% in the North Plains and Pacific Northwest to 84.6% in the High Plains (Herrick et al., 2022). Seasonally, LA prevalence is reported to be less in January and greater in late spring/early summer (e.g., April to June; Grimes et al., 2024). In the current study, cattle were harvested from late January to early February. Mean liver *Salmonella* prevalence was not affected by the presence of LA. Likewise, Dockray (2022) did not find a difference in *Salmonella* prevalence or concentration between healthy and abscessed livers collected quarterly from commercial beef processing plants in the High Plains region. Although seasonality could potentially explain the decreased *Salmonella* prevalence observed in the current study compared with regional means, further studies are warranted to elucidate the effects of regionality and seasonality on *Salmonella* prevalence in healthy and abscessed livers.

### 4.2 *Fusobacterium necrophorum* subsp. *necrophorum*, subsp. *funduliforme*, and *F. varium* in ruminal fluid, feces, and liver

Until recently, *F. necrophorum* enumeration in ruminal contents relied on culture-dependent methods that incorporated selective growth medium containing lactate as the primary carbon source and indole as a growth indicator (Tan et al., 1994); however, this methodology to quantify *F. necrophorum* unintentionally inflated cell densities because of similar fermentative mechanisms shared with *F.* var*ium* (Schwarz et al., 2023). Therefore, it is likely that for many years *F. necrophorum* subsp. *necrophorum* has been misidentified from culture methods (Schwarz et al., 2023; Deters et al., 2024a). The recent development of a qPCR assay (Deters et al., 2024a) to detect and quantify *F. necrophorum* subsp. *necrophorum*, subsp. *funduliforme,* and *F. varium* has greatly improved identification and enumeration of these bacterial species across different sample types.

We noted that *F. necrophorum* subsp. *necrophorum* and subsp. *funduliforme* abundance in ruminal fluid were not indicative of LA presence. Although in agreement with Deters et al. (2024a), samples in that study were collected once at harvest, not longitudinally before harvest as in the current study. Unlike Deters et al. (2024a), *F. varium* abundance in ruminal fluid in the current study was greater in cattle with LA. Nonetheless, the role of *F. varium* in LA development is still yet to be fully understood. Although *F. varium* is considered actively invasive (Manson McGuire et al., 2014), it lacks the leukotoxin gene found in *F. necrophorum* that induces abscess development (Narayanan et al., 2001). Unlike Schwarz et al. (2023), *F. varium* was not the dominant *Fusobacterium* species in ruminal fluid in the current study. Differences in sample collection day relative to harvest, feedlot regionality, and sample analysis (i.e., absolute vs. relative abundance) and processing (i.e., samples were not enriched prior to qPCR analysis in the current study) makes a direct comparison between studies difficult. Nevertheless, *F. varium* has been associated with human infections and diseases such as ulcerative colitis and acute kidney failure (Minami et al., 2009; Lee et al., 2022), justifying further research to understand its pathogenicity and risk as a potential zoonotic cattle pathogen.

From feedlot arrival to harvest, subsp. *necrophorum* and *F. varium* prevalence in ruminal fluid increased, a response that could be associated with long-term feeding of high-concentrate diets. *Fusobacterium necrophorum* can use lactate as a carbon and energy source, and increasing the proportion of grain in finishing cattle diets increases ruminal lactate production (Monteiro and Faciola, 2020) and *F. necrophorum* concentrations as well (Tan et al., 1996). Although it has been speculated whether *F. varium* uses lactate as an energy source, the presence of a lactate dehydrogenase gene in the *F. varium* genome highlights potential overlapping metabolic pathways and similar ecological niches with *F. necrophorum* (Schwarz et al., 2023).

At harvest, *F. necrophorum* subsp. *necrophorum* and *F. varium* prevalence in ruminal fluid was 93.3 and 71.2%, respectively, regardless of the LA status (Figures 2A,C). Deters et al. (2024a) reported *F. necrophorum* subsp. *necrophorum* was more prevalent in the ruminal contents of cattle with LA than without; however, mean subsp. *necrophorum* prevalence was less than 29% regardless of LA presence. This lead Deters et al. (2024a) to suggest that *F. necrophorum* subsp. *necrophorum* is not a normal inhabitant of the rumen. The data presented herein contradict that suggestion and agree with previous reports (Langworth, 1977; Wada, 1978; Smith and Thornton, 1993; Tadepalli et al., 2009) that infer subsp. *necrophorum* is a normal inhabitant of the rumen. *Fusobacterium necrophorum* subsp. *funduliforme* was present in 100% of ruminal fluid samples at harvest in the current study (Figure 2B). Deters et al. (2024a) reported *F. necrophorum* subsp. *funduliforme* prevalence was over 90% in ruminal content regardless of the LA status, therein validating subsp. *funduliforme* as a normal inhabitant of the rumen. Of note, ruminal fluid in the current study was collected from cattle longitudinally at 1 feedlot in the High Plains while Deters et al. (2024a) collected ruminal fluid from cattle originating from 12 feedlots at a Midwest commercial beef abattoir, and a better understanding of regional influences on ruminal *F. necrophorum* populations could prove worthwhile. Though ruminal fluid was not collected, Herrick et al. (2022) reported the incidence of *F. necrophorum* subsp. *necrophorum* and subsp. *funduliforme* in fed beef livers differed across regions of the U.S., suggesting a potential effect of region currently exists. Additionally, cross-sectional data from Deters et al. (2024a) was gathered from a single collection timepoint, thereby limiting the ability to assess temporal changes associated with dietary transition and disease progression. In contrast, the present study used a longitudinal approach to track shifts in *Fusobacterium* and *Salmonella* populations within individual animals over time in response to feedlot management.

Despite the belief that fecal excretion of *F. necrophorum* is the primary source of foot rot, the presence of *F. necrophorum* in feces is rare (Nagaraja et al., 2005). Smith and Thornton (1993) reported 2.5% (2/81) of calves sampled on a farm experiencing necrobacillosis were fecal positive for *F. necrophorum* biovar A (i.e., subsp. *necrophorum*). This led the authors to conclude that a surprisingly small proportion of cattle were excreting *F. necrophorum* despite high ruminal prevalence (83%). Moreover, neither fecal nor soil *Fusobacterium* were selected for model inclusion when estimating the LA occurrence within pens (Weinroth et al., 2019). In English sheep farms, Clifton et al. (2019) reported *F. necrophorum* was not ubiquitous in soil, and was only cultured from the surface of wet, highly-trafficked areas. This suggests *F. necrophorum* contamination of soil is transient. Kilama et al. (2024) reported that bull feces were negative for *F. necrophorum* subsp. *necrophorum,* subsp. *funduliforme,* and *F. varium* when assayed using qPCR; however, 12.5% of fecal samples were *F. varium* positive following enrichment. In the current study, fecal *F. necrophorum* subsp. *necrophorum* and subsp. *funduliforme* prevalence did not differ from feedlot arrival to harvest and were not associated with LA presence (Figures 3A,B). Jennings et al. (2021) reported colonic *F. necrophorum* subsp. *necrophorum* prevalence ranged from 0 to 19.6% over a 231-d feeding trial, with prevalence greatest on d 112. Mean fecal prevalence of *F. necrophorum* subsp. *necrophorum* in the current study was 16.8%. The increased prevalence of *F.* var*ium* from d 35 to harvest is potentially associated with increased ruminal prevalence over the same timeframe (Figure 3C). Nonetheless, the lack of a similar response in *F. necrophorum* subsp. *necrophorum* warrants further investigation. Fecal *Fusobacterium* abundance in the current study was low and not altered by collection day or LA prevalence. Previously, Kim and Wells (2016) assigned 7 bovine fecal microbiome sequences out of 13,663 to *Fusobacteria* (0.0005%; Kim and Wells, 2016), further suggesting the lower GIT is an unfavorable environment for *Fusobacterium* survival and proliferation.

The abundance of *F. necrophorum* subsp. *necrophorum* in abscessed liver tissue was greater than in healthy liver tissue. This was expected and in agreement with previous reports (Stotz et al., 2021; McDaniel et al., 2024b). An unexpected response, however, was the lack of difference in *F. necrophorum* subsp. *necrophorum* prevalence between healthy and abscessed liver tissue. Even though earlier studies have documented *F. necrophorum* subsp. *necrophorum* in healthy liver tissue (Stotz et al., 2021; McDaniel et al., 2024a, 2024b), the prevalence of subsp. *necrophorum* in LA in the current study (54.8%) is less than the reported average of 79.3% for fed beef steers (Herrick et al., 2022). Potential reasons for this disparity include study scale (i.e., feedlot specific vs. national) and that half the liver samples received at LIRU from cattle recorded to have LA did not have a physical abscess in the processed sample. As a result, it is possible the abundance and prevalence reported in the current study is an underestimation.

When rumenitis or intestinal barrier dysfunction occurs, the translocation of gut bacteria and pathogens into portal circulation is not necessarily selective. Hence, it could be expected that greater ruminal fluid prevalence of *F. necrophorum* subsp. *funduliforme* will lead to greater liver prevalence of subsp. *funduliforme* than subsp. *necrophorum*. Nevertheless, prevalence of subsp. *funduliforme* in LA was 42.9% in the current study. Herrick et al. (2022) reported subsp. *funduliforme* prevalence to range from 15.4 to 44.0% in LA, with prevalence being greatest in the Pacific Northwest and lesser in the High Plains and Desert Southwest. Moreover, regardless of region, subsp. *funduliforme* prevalence in LA was not associated with subsp. *necrophorum* or *Salmonella* prevalence in LA (Herrick et al., 2022). Lesser subsp. *funduliforme* prevalence in LA is likely attributable to the weaker promoter associated with the *lktA* operon in *F. necrophorum* subsp. *funduliforme*, thereby inferring less virulence when compared with subsp. *necrophorum*. The leukotoxin operon in *F. necrophorum* subsp. *necrophorum* and subsp. *funduliforme* is tricistronic and comprised of *lktB*, *lktA*, and *lktC* genes. Work by Tan et al. (1992) noted *F. necrophorum* subsp. *funduliforme* leukotoxin specific mRNA expression was 18-fold less than subsp. *necrophorum*. Tadepalli et al. (2008) later validated this competitive disadvantage, reporting a 21-fold decrease in gene expression of *lktA* in *F. necrophorum* subsp. *funduliforme*.

The abundance of *F.* var*ium* in livers was low, with prevalence ranging from 4.8 to 7.1% for healthy and abscessed livers, respectively. Deters et al. (2024b) reported qPCR prevalence of *F. varium* in LA to be 1% before enrichment and 10.4% after enrichment. As *F. varium* does not contain the leukotoxin gene found in *F. necrophorum* subsp. *necrophorum* and subsp. *funduliforme* to evade host defense mechanisms, it is not surprising *F. varium* abundance in the liver is the lowest of the 3 *Fusobacterium* quantified. Since *F. varium* is considered actively invasive, it is likely that a portion of *F. varium* will inevitably reach the liver (Schwarz et al., 2023); however, whether this pathogenesis aids in the ability for subsp. *necrophorum* to enter portal circulation has yet to be demonstrated.

## 5 Conclusion

The results of this study provide important insights into the dynamics of *Fusobacterium* and *Salmonella* populations within the GIT of feedlot cattle with and without LA. While direct correlations between bacterial populations and LA presence were not observed, the findings herein highlight the complexity of factors influencing pathogen persistence in the GIT. For instance, the observed differences in fecal *Salmonella* concentration and prevalence between steers with and without LA suggest that gut barrier function may influence the risk of LA development. Moreover, the transition to a high-concentrate diet appears to create an unfavorable environment in the rumen that limits *Salmonella* persistence but enhances the proliferation of *F. necrophorum* subsp. *necrophorum* and *F. varium* regardless of LA presence. Thus, high-concentrate feedlot diets potentiate the risk of a *Fusobacterium* infection in the rumen, while facilitating *Salmonella* persistence in the lower GIT with greater days on feed. Although current results suggest *Fusobacterium* species are normal inhabitants of the ruminal microbiome in feedlot cattle, fecal *Fusobacterium* abundance and prevalence is low. Nevertheless, *Fusobacterium* were prevalent in both healthy and abscessed livers, with subsp. *necrophorum* abundance being greater in abscessed liver tissue. In conclusion, entry of *Fusobacteria* and *Salmonella* into portal circulation is possible throughout the GIT though the abundance and prevalence of these bacterial populations are not directly suggestive of LA formation. These results underscore the need for further investigation into the complex interactions between host immunity, gut microbiome dynamics, and pathogen colonization. Future research should focus on how dietary transitions affect microbial communities in modulating *Fusobacterium* and *Salmonella* populations in feedlot cattle. Additionally, studies investigating the effects of feedlot health management practices on gut epithelial integrity and LA formation in feedlot cattle will aid in understanding the broader factors influencing LA susceptibility and progression.

## Data Availability

The raw data supporting the conclusions of this article will be made available by the authors, without undue reservation.
